# Necrosis correlates with high vascular density and focal macrophage infiltration in invasive carcinoma of the breast

**DOI:** 10.1038/sj.bjc.6690158

**Published:** 1999-02

**Authors:** R D Leek, R J Landers2, A L Harris, C E Lewis

**Affiliations:** ICRF Molecular Oncology Laboratory, University of Oxford, Institute of Molecular Medicine, John Radcliffe Hospital, Oxford, OX3 9DU, UK; Department of Pathology, University of Sheffield Medical School, Beech Hill Road, Sheffield, SE10 2JF, UK

**Keywords:** necrosis, hypoxia, angiogenesis, macrophage, breast cancer

## Abstract

Necrosis is a common feature of invasive carcinoma of the breast and is caused by chronic ischaemia leading to infarction. Although necrosis was previously assumed to be due to a generally poor blood supply in the tumour, in this study we show that it is present in tumours with focal areas of high vascular density situated away from the actual sites of necrosis. This may account, in part, for the previous observation that necrosis is linked to poor prognosis in this disease. Highly angiogenic tumours often display blood vessel shunting from one tumour area to another, which further exacerbates ischaemia and the formation of tumour necrosis. We have recently demonstrated that high focal microphage infiltration into breast tumours is significantly associated with increased tumour angiogenesis and poor prognosis and that the macrophages accumulate in poorly vascularized, hypoxic areas within breast tumours. In order to investigate the interactions of macrophages with chronic ischaemia (as reflected by the presence of necrosis) and angiogenesis in breast tumours, we quantified the levels of these three biological parameters in a series of 109 consecutive invasive breast carcinomas. We found that the degree of tumour necrosis was correlated with both microphage infiltration (Mann–Whitney *U*, *P*-value = 0.0009; chi-square, *P*-value = 0.01) and angiogenesis (Mann–Whitney *U P*-value = 0.0008, chi square *P*-value = 0.03). It was also observed that necrosis was a feature of tumours possessing an aggressive phenotype, i.e. high tumour grade (chi-square, *P*-value < 0.001), larger size (Mann–Whitney *U*, *P*-value = 0.003) and low oestrogen receptor status (Mann–Whitney *U*, *P*-value = 0.008; chi-square, *P*-value < 0.008). We suggest, therefore, that aggressive tumours rapidly outgrow their vascular supply in certain areas, leading to areas of prolonged hypoxia within the tumour and, subsequently, to necrosis. This, in turn, may attract macrophages into the tumour, which then contribute to the angiogenic process, giving rise to an association between high levels of angiogenesis and extensive necrosis. © 1999 Cancer Research Campaign


*British Journal of Cancer* (1999) **79**(5/6), 991–995
© 1999 Cancer Research Campaign
Article no. bjoc.1998.0158

### Necrosis correlates with high vascular density and focal macrophage infiltration in invasive carcinoma of the breast

**RD Leek[1,*], RJ Landers[2*], AL Harris[1] and CE Lewis[2]**

[1]ICRF Molecular Oncology Laboratory, University of Oxford, Institute of Molecular Medicine, John Radcliffe Hospital, Oxford OX3 9DU, UK; [2]Department of Pathology, University of Sheffield Medical School, Beech Hill Road, Sheffield SE10 2JF, UK

**Summary** Necrosis is a common feature of invasive carcinoma of the breast and is caused by chronic ischaemia leading to infarction. Although necrosis was previously assumed to be due to a generally poor blood supply in the tumour, in this study we show that it is present in tumours with focal areas of high vascular density situated away from the actual sites of necrosis. This may account, in part, for the previous observation that necrosis is linked to poor prognosis in this disease. Highly angiogenic tumours often display blood vessel shunting from one tumour area to another, which further exacerbates ischaemia and the formation of tumour necrosis. We have recently demonstrated that high focal microphage infiltration into breast tumours is significantly associated with increased tumour angiogenesis and poor prognosis and that the macrophages accumulate in poorly vascularized, hypoxic areas within breast tumours. In order to investigate the interactions of macrophages with chronic ischaemia (as reflected by the presence of necrosis) and angiogenesis in breast tumours, we quantified the levels of these three biological parameters in a series of 109 consecutive invasive breast carcinomas. We found that the degree of tumour necrosis was correlated with both microphage infiltration (Mann–Whitney *U*, *P*-value = 0.0009; chi-square, *P*-value = 0.01) and angiogenesis (Mann–Whitney *U P*-value = 0.0008, chi square *P*-value = 0.03). It was also observed that necrosis was a feature of tumours possessing an aggressive phenotype, i.e. high tumour grade (chi-square, *P*-value < 0.001), larger size (Mann–Whitney *U*, *P*-value = 0.003) and low oestrogen receptor status (Mann–Whitney *U*, *P*-value = 0.008; chi-square, *P*-value < 0.008). We suggest, therefore, that aggressive tumours rapidly outgrow their vascular supply in certain areas, leading to areas of prolonged hypoxia within the tumour and, subsequently, to necrosis. This, in turn, may attract macrophages into the tumour, which then contribute to the angiogenic process, giving rise to an association between high levels of angiogenesis and extensive necrosis.

**Keywords:** necrosis; hypoxia; angiogenesis; macrophage; breast cancer

Central necrosis is a common feature in invasive breast cancer and is associated with poor outcome and tumour aggression. For example, findings from the National Surgical Adjuvant Breast Project showed that, after 10 years' follow-up, the presence of moderate to marked central tumour necrosis was associated with reduced relapse-free survival and increased mortality in both node-positive and node-negative patients (Fisher et al, 1993) with invasive breast carcinoma. The term necrosis may be defined as the morphological changes following cell death, and may be coagulative where the tissue is solid and the original structures are largely preserved. In this case, the autolytic enzymes are inactive and the cells within an area of coagulative necrosis retain their shape but are highly eosinophilic 'ghosts' of their previous form. After a period of time, this pattern is replaced by liquefaction necrosis, in which the cellular structures are broken down by proteolytic enzymes released from ruptured lysosomes and similar enzymes released by infiltrating inflammatory cells (Woolf, 1986). It is thought that tumour necrosis is caused by chronic ischaemia (i.e. hypoxia, low pH, low glucose, high lactate) within tumours, caused by vascular collapse, blood shunting to other sites and/or rapid tumour cell growth overtaking the rate of neovascularization in a given area.

The involvement of hypoxia in tumour progression, and especially tumour angiogenesis, has become a prominent area of research in recent times. Moreover, the degree of tumour hypoxia has been inversely correlated with response to radio- and various chemotherapies and overall survival (reviewed by Dachs et al, 1996). Angiogenesis is a multistep process involving complex cellular interactions between endothelial cells and malignant epithelial cells, stromal cells and the tumour matrix. The process of angiogenesis is distinct from that of vasculogenesis, in that it is the process of vessel formation derived from existing vasculature. A number of studies have shown that it is an essential requirement for tumour growth and metastasis (Blood and Zetter, 1990), and that hypoxia may be a primary driving force behind angiogenesis in malignant tumours.

Hypoxia up-regulates the expression of a number of angiogenic growth factors, and the hypoxia-inducible transcription factor, hif-1$\alpha$, has been found to be involved in the up-regulation of a number of hypoxia-adaptive mechanisms within tumour cells. These include up-regulation of glycolytic enzymes such as lactate dehydrogenase and phosphoglycerol kinase (which help tumour cells to switch to anaerobic glycolysis in hypoxia) (Semenza et al, 1994), as well as the angiogenic growth factor vascular endothelial

*Received 3 April 1998*
*Revised 8 July 1998*
*Accepted 14 July 1998*

*Correspondence to:* CE Lewis

*The first two authors contributed equally to this study.

growth factor (VEGF) (Forsythe et al, 1996). Hypoxia may also regulate angiogenesis indirectly via its effect on tumour-associated macrophages (TAMs). We have recently demonstrated an association between focal TAM infiltration and reduced relapse-free and overall survival in breast carcinomas. We have also shown that high levels of macrophages in focal areas (MØI) are associated with increased vascular density and that macrophage clusters are found in avascular areas of breast tumours (Leek et al, 1997). As macrophages have been shown to release such proangiogenic cytokines as vascular endothelial growth factor (Harney et al, 1998) tumour recrosis factor α (TNF-α) in response to hypoxia (Scannell et al, 1993), it is possible that, once macrophages reach hypoxic/ischaemic tumour sites, they may promote tumour growth and metastasis by releasing these factors to stimulate angiogenesis. Therefore, the purpose of this study was to assess quantitatively the degree of central necrosis in a consecutive series of invasive breast carcinomas and to examine its relationship to both focal microphage infiltration and angiogenesis, as measured by quantitative CD68 and CD31 immunohistochemistry of vessel and macrophage 'hotspots' respectively (Fox et al, 1995; Leek et al, 1996, 1997).

### MATERIALS AND METHODS

### Tumours and patients

A consecutive series of 109 surgically resected invasive breast carcinomas were retrieved from the archives of the John Radcliffe Hospital, Oxford, UK. Surgery had been performed between 1989 and 1992, all had undergone axillary node sampling, and the presence of nodal metastasis was confirmed histologically. The modified Bloom and Richardson method was used to grade all invasive carcinomas of ductal type (Elston, 1987), and all patients were followed up every 3 months for the first 18 months and every 6 months thereafter. The characteristics of this series of tumours are detailed in Table 1. All patients received either simple mastectomy or lumpectomy and radiotherapy; adjutant radiotherapy was administered to the ipsilateral axilla if histological evidence of nodal metastasis was found. Patients with confirmed recurrent disease were treated by endocrine manipulation for soft tissue or skeletal disease or by chemotherapy for visceral disease or failed endocrine therapy. Patients with isolated soft-tissue relapse received radiotherapy. Details of adjuvant treatment consisting of tamoxifen for 5 years and cyclophosphamide, methotrexate and 5-fluorouracil (CMF) intravenously for six courses are shown in Table 1.

### Assessment of tumour necrosis

Necrosis was subjectively graded into three categories using routinely processed haematoxylin and eosin-stained histological sections. 0 = no necrosis, 1 = focal areas of necrosis (<25% of tumour), 2 = widespread areas of necrosis (25–75% of tumour) and 3 = virtually all necrosis (75–100% of tumour). For cut-off point analyses the presence of any necrosis was considered positive.

### Tumour hormone receptor status

Oestrogen receptor (ER) analysis was performed using an enzyme-linked immunosorbent assay (ELISA) technique (Abbott Laboratories, USA). Tumours with cytoplasmic oestrogen levels higher than 5 fmol $mg^{-1}$ protein were considered positive (EORTC Breast Cancer Co-operative Group, 1980). Epidermal growth factor receptor (EGFR) was determined using ligand binding of [$^{125}$I]EGF to tumour membranes. Tumours with an EGFR level of greater than 20 fmol $mg^{-1}$ protein were considered positive (Nicholson et al, 1988).

**Table 1** Clinical and pathological features of tumours and patients

| Patient characteristics | Number |
|---|---|
| Age (median, range) years | 55 (28–83) |
| <50 | 42 |
| ≥50 | 67 |
| Surgical treatment | |
| Lumpectomy + RT | 95 |
| Mastectomy | 14 |
| Adjuvant treatment | |
| Chemotherapy | 29 |
| Tamoxifen | 50 |
| Lymph node status | |
| Negative/positive | 72/37 |
| Tumour size (median, range) cm | 2.3 (1–7) |
| <2 | 36 |
| ≥2 | 57 |
| Histology | |
| Ductal | 88 |
| Lobular | 8 |
| Others | 13 |
| Grade | |
| I | 9 |
| II | 43 |
| III | 36 |
| ER[a] (median, range) | 9.7 (0–695) |
| <10 | 56 |
| ≥10 | 53 |
| EGFR[a] (median, range) | 18.2 (0–710) |
| <20 | 58 |
| ≥20 | 49 |
| Survival follow-up (median, range) months | 63 (1.7–93) |
| Deaths, recurrences | 31, 37 |

[a]fmol $mg^{-1}$ protein.

### Vascular grade and macrophage index

Chalkley vascular count (CVC) and focal macrophage infiltration (MØI) were quantitatively determined using Chalkley point array counting methodologies described by us and others previously (Fox et al, 1995; Leek et al, 1996). Vessels are stained immunohistochemically using an antibody against CD31, and macrophages are stained with anti-CD68. Using this system, CVC and MØI are determined microscopically by enumerating the numbers of either vessels or macrophages in the three areas of the section at high power (×25 objective) which show the most dense staining, the so called 'hotspot' areas. For cut-off point analysis the medians were used to categorize tumours into high and low groups (high CVC ≥7, high MØI ≥12).

### Statistical analysis

Chi-square and Fisher's exact tests were used to investigate relationships between categorical tumour variables, and Mann–Whitney

 © Cancer Research Campaign 1999

**Table 2** Summary of chi-square tests showing associations between necrosis and other tumour variables

| | Observed frequencies | | | | |
|---|---|---|---|---|---|
| | Grade | | | ER[a] | |
| | 1 | 2 | 3 | ER negative | ER positive |
| Necrosis | 0 | 14 | 25 | 31 | 15 |
| No necrosis | 9 | 29 | 11 | 25 | 38 |
| Chi-square | | 18.7 | | 8.2 | |
| Chi-square *P*-value | | <0.001 | | <0.008 | |

**Table 3** Summary of chi-square tests showing associations between necrosis and angiogenesis and macrophage infiltration

| | Observed frequencies | | | |
|---|---|---|---|---|
| | Vascular density | | Macrophage index | |
| | High CVC | Low CVC | High MØI | Low MØI |
| Necrosis | 17 | 29 | 37 | 9 |
| No necrosis | 10 | 51 | 34 | 27 |
| Chi-square | 5.9 | | 7.2 | |
| Chi-square *P*-value | 0.03 | | 0.01 | |

non-parametric tests were utilized to compare categorical with continuous tumour variables. These analyses were performed using Statview 4.5 statistical analysis software (Abacus concepts, Berkeley, CA, USA). Survival analysis was performed using the log-rank test to evaluate differences between life tables. Survival analyses were accomplished using Stata release 3.1 software (Stata Corp., College Station, TX, USA).

### RESULTS

### Distribution of necrosis grading

In this series of 109 breast cancers, 29 (26.6%) cases were necrosis grade 1, 16 (14.7%) cases necrosis grade 2 and only one case was grade 3 (0.9%). This gave a total of 46 cases with necrosis present (42.2%) compared with 63 with no necrosis (57.8%). For this reason it was decided, for the purposes of statistical analysis, to split the data into two groups of those with and those without necrosis.

### Relationship of necrosis to clinicopathological tumour variables

Tumour necrosis was compared with tumour variables known to relate to outcome, including node status, size of tumour at excision, patient age, histological type, grade, ER expression and EGFR expression. A number of significant associations were observed, including positive associations between the presence of necrosis with tumour size (Mann–Whitney $U$, $P = 0.003$) and increasing age of patient (Mann–Whitney $U$, $P = 0.02$), when these data were examined as continuous variables. An association was also seen between increasing necrosis grade and high tumour grade (chi square, $P = 0.0001$) (Table 2). When examining ER and EGFR, an inverse association was observed between ER expression and necrosis (Mann–Whitney $U$, $P = 0.008$; chi square, $P = 0.007$) (Table 2). Conversely, a positive association was noted between EGFR and the degree of necrosis when using the lower necrosis cut-off point (Mann–Whitney $U$, $P = 0.047$). No associations were seen between necrosis and node status or histological type.

### Relationship of necrosis to vascular count and focal macrophage infiltration

When tumour necrosis was compared with vascular count and macrophage index, significant associations were observed with both variables (see Table 3 for summary). An association was noted between higher vascular density and increasing necrosis (Mann–Whitney $U$, $P = 0.008$; chi-square, $P = 0.03$). Also, significant positive associations were observed between increasing macrophage index and the degree of necrosis (Mann–Whitney $U$, $P = 0.0009$; chi-square, $P = 0.01$), with high numbers of TAMs often seen to lie within the palisading region around areas of necrosis.

### Necrosis and prognosis

No effect was observed on either relapse-free (RFS) or overall survival (OS) by the presence of necrosis [log-rank (Mantel–Cox): RFS, $P = 0.27$; OS, $P = 0.87$] or high levels of necrosis [log-rank (Mantel–Cox); RFS, $P = 0.56$; OS, $P = 0.82$].

### DISCUSSION

Tumour necrosis is generally thought to result from rapidly proliferating tumour cells outpacing their blood supply in certain areas. This causes areas within the tumour which are poorly vascularized to become ischaemic. If this condition is sustained in these regions, the result is the onset of necrosis. In this study of breast

© Cancer Research Campaign 1999

carcinomas, we found the presence of tumour necrosis to be related to higher levels of angiogenesis, as measured by increased vascular density in focal areas. Highly necrotic tumours also contained higher levels of focal macrophage infiltration. These results accord well with other previous, albeit only semiquantitative, reports in which simply the presence of necrosis in breast carcinomas was correlated with (1) decreased survival and total vessel counts in highly vascularized tumour areas (Kato et al, 1997) and (2) diffuse tumour infiltration by macrophages (Lee et al, 1997). However, neither of these studies attempted to quantify the actual amount of necrosis present. Furthermore, Lee et al (1997) only loosely assessed the general level of TAM infiltration per tumour. In the present study, we have used previously published methods to quantify the degree of necrosis as well as the highest density of vessels and macrophages in defined areas of the tumour where ‘hotspots’ of these cells occur. These parameters were chosen, rather than an assessment of the total number of vessels or macrophages across the tumour as a whole, as we have previously shown that these correlate with such important indices of tumour aggressivity such as lymph node status and/or prognosis (Fox et al, 1995; Leek et al, 1996, 1997).

The finding that higher levels of necrosis were present in more angiogenic tumours may appear at first to be counterintuitive. However, a contributing factor to tumour progression is angiogenesis, and increased angiogenesis in certain areas leads to rapid growth of the tumour cells at those sites. This will lead to the development of focal areas of ischaemia within such fast-growing tumours as neoplastic cell proliferation follows and then outstrips angiogenic induction. This assertion is also supported by the fact that necrosis is associated with larger tumours and those of high grade as well as increasing EGFR, and is inversely associated with ER expression, indicating that necrosis is a feature of tumours possessing an aggressive phenotype. It is interesting to note that the levels of tumour necrosis appear to drop with increasing age of the patient, indicating once again that necrosis may be correlated with tumour aggressivity as breast tumours in younger patients are often of a more aggressive nature.

In addition, high levels of angiogenesis may not actually correspond to improved blood flow within tumours as the new blood vessels are incomplete and often lack structural integrity (Blood and Zetter, 1990). They may collapse and block flow or they may not be capable of containing arterial pressure within their structure, leading to localized haemorrhage. Anastomosis of new vessels may also lead to shunting of the blood flow, resulting in poor flow in vessels efferent to shunts (Helmlinger et al, 1997). The findings of this study suggest that tumour ischaemia may be an important factor in the stimulus of tumour growth. It is well known that a number of angiogenic factors are up-regulated by hypoxia (Dachs et al, 1996), including VEGF (Forsythe et al, 1996), and that hypoxic tumour cells may, therefore, stimulate angiogenesis directly via their release of such factors. Indeed, Plate and Warnke (1997) and others have shown the up-regulation of angiogenic factor expression around areas of necrosis in breast carcinomas.

Our finding that increased necrosis was associated with higher levels of focal macrophage infiltration, and that a high number of macrophages cluster around necrotic areas, matches a similar report by Negus et al (1997) in malignant ovarian tumours. We have previously demonstrated that in breast cancer increased MØI is associated with reduced overall and relapse-free survival, and is also associated with increased angiogenesis. We have also shown that TAM hotspots are located in avascular areas of tumours, suggesting that they preferentially migrate towards areas of relative hypoxia (Leek et al, 1997). Indeed, when macrophages infiltrate into multicell tumour spheroids in vitro, they accumulate rapidly in and around the hypoxic and necrotic central area of these three-dimensional cultures of tumour cells (Lewis, 1996). We have, therefore, suggested that the TAM population of tumours may be important contributors to the stimulation of tumour angiogenesis, as they are also capable of producing a large repertoire of angiogenic factors, including angiogenic cytokines such as VEGF and TNF-α, and proteolytic enzymes (Lewis et al, 1995; Leek et al, 1997, 1998). Indeed, it is our intention to examine expression of such factors by macrophages, both in human tumours and in experimental models. It would seem that macrophages are attracted to necrotic tumours, perhaps by chemotactic factors released by dead/dying or hypoxic tumour cells, such as VEGF, which is chemotactic for macrophages (Clauss et al, 1990).

The results from this study show that necrosis, resulting from chronic ischaemia, is associated with high rather than low angiogenesis. As macrophage infiltration is also elevated in these more necrotic, highly angiogenic tumours, we suggest that macrophages may play a key role in the aggressive tumour phenotype, in part by promoting tumour angiogenesis.

### REFERENCES

Blood CH and Zetter BR (1990) Tumor interactions with the vasculature: angiogenesis and tumor metastasis. *Biochim Biophys Acta* **1032**: 89–118

Clauss M, Gerlach M, Gerlach H, Brett J, Wang F, Familletti PC, Pan YC, Olander JV, Connolly DT and Stern D (1990) Vascular permeability factor: a tumor-derived polypeptide that induces endothelial cell and monocyte procoagulant activity, and promotes monocyte migration. *J Exp Med* **172**: 1535–1545

Dachs GU and Stratford IJ (1996) The molecular response of mammalian-cells to hypoxia and the potential for exploitation in cancer therapy. *Br J Cancer* **74**: S126–S132

Elston CW (1987) Grading of invasive breast carcinomas. In *Diagnostic Histopathology of the Breast*, Page D and Anderson T (eds). Churchill Livingstone: Edinburgh

European Breast Cancer Co-operative Group (1980) Revision of the standards for the assessment of hormone receptors in human breast cancer; report of the second EORTC Workshop, held on 16–17 March 1979, the Netherlands Cancer Institute. *Eur J Cancer* **16**: 1513–1513

Fisher ER, Anderson S, Redmond C and Fisher B (1993) Pathologic findings from the National Surgical Adjuvant Breast Project protocol B-06. 10-year pathologic and clinical prognostic discriminants. *Cancer* **71**: 2507–2514

Forsythe JA, Jiang BH, Iyer NV, Agani F, Leung SW, Koos RD and Semenza GL (1996) Activation of vascular endothelial growth-factor gene-transcription by hypoxia-inducible factor-1. *Mol Cell Biol* **16**: 4604–4613

Fox SB, Leek RD, Weekes MP, Whitehouse RM, Gatter KC and Harris AL (1995) Quantitation and prognostic value of breast cancer angiogenesis: comparison of microvessel density, Chalkley count, and computer image analysis. *J Pathol* **177**: 275–283

Harney JH, Dimitriadis E, Kay E, Redmond HP and Bouchier-Hayes D (1998) Regulation of macrophage production of VEGF by hypoxia and $TGF_1$ 3-1. *Ann Surg Oncol* **5**: 271–278

Helmlinger G, Yuar F, Dellian M and Jain RK (1997) Interstitial pH and pO2 gradients in solid tumors in vivo: high-resolution measurements reveal a lack of correlation. *Nature Med* **3**: 177–182

Kato T, Kimura T, Miyakawa R, Tanaka S, Fuji A, Yamamoto K, Kameoka S, Hamano K, Kawakami M and Aiba M (1997) Clinicopathologic study of angiogenesis in Japanese patients with breast cancer. *World J Surg* **21**: 49–56

Lee AHS, Happerfield LC, Bobrow LG and Millis RR (1997) Angiogenesis and inflammation in invasive carcinoma of the breast. *J Clin Pathol* **50**: 669–673

Leek RD, Lewis CE, Whitehouse R, Greenall M, Clarke J and Harris AL (1996) Association of macrophage infiltration with angiogenesis and prognosis in invasive breast-carcinoma. *Cancer Res* **56**: 4625–4629

© Cancer Research Campaign 1999

Leek RD, Lewis CE and Harris AL (1997) The role of macrophages in tumour angiogenesis. In *Tumour Angiogenesis*, Bicknell R, Lewis CE and Ferrara N (eds), Oxford University Press: Oxford

Leek RD, Landers RJ, Fox SB, Ng F, Harris AL and Lewis CE (1998) Association of tumour necrosis factor alpha and its receptors with thymidine phosphorylase expression and metastasis in invasive carcinoma of the breast. *Br J Cancer* **77**: 2246–2251

Lewis CE, Leek RD, Harris AL and McGee JOD (1995) Cytokine reguation of angiogenesis in breast cancer: the role of tumor-associated macrophages. *J Leukoc Biol* **57**: 747–751

Lewis CE (1996) Regulation of tumour angiogenesis by macrophages: role of hypoxia and angiogenic factor production. *Clin Exp Met* **15**: 74

Negus RPM, Stamp GWH, Hadley J and Balkwill FR (1997) Quantitative assessment of the leukocyte infiltrate in ovarian cancer and its relationship to the expression of C-C chemokines. *Am J Pathol* **150**: 1723–1734

Nicholson S, Sainsbury JR, Needham GK, Chambers P, Farndon JR and Harris AL (1988) Quantitative assays of epidermal growth factor receptor in human breast cancer: cut-off points of clinical relevance. *Int J Cancer* **42**: 36–41

Plate KH and Warnke PC (1997) Vascular endothelial growth factor. *J Neurooncol* **35**: 365–372

Scannell G, Waxman K, Kaml GJ, Ioli G, Gatanaga T, Yamamoto R and Granger GA (1993) Hypoxia induces a human macrophage cell line to release tumour necrosis factor-alpha and its soluble receptors in vitro. *J Surg Res* **54**: 281–285

Semenza GL, Roth PH, Fang HM and Wang GL (1994) Transcriptional regulation of genes encoding glycolytic enzymes by hypoxia-inducible factor 1. *J Biol Chem* **269**: 23757–23763

Woolf N (1986) Cell and tissue death. In *Cell, Tissue and Disease, The Basis of Pathology*. pp. 25–30 Baillière Tindall: London

© Cancer Research Campaign 1999
